# An Efficient Strategy for Broad-Range Detection of Low Abundance Bacteria without DNA Decontamination of PCR Reagents

**DOI:** 10.1371/journal.pone.0020303

**Published:** 2011-05-26

**Authors:** Shy-Shin Chang, Hsung-Ling Hsu, Ju-Chien Cheng, Ching-Ping Tseng

**Affiliations:** 1 Graduate Institute of Clinical Medical Sciences, Department of Medicine, College of Medicine, Chang Gung University, Tao-Yuan, Taiwan, Republic of China; 2 Department of Medicine, College of Medicine, Chang Gung University, Tao-Yuan, Taiwan, Republic of China; 3 Department of Family Medicine and Emergency Medicine, Chang Gung Memorial Hospital, Tao-Yuan, Taiwan, Republic of China; 4 Department of Medical Biotechnology and Laboratory Science, Chang Gung University, Tao-Yuan, Taiwan, Republic of China; 5 Department of Medical Laboratory Science and Biotechnology, China Medical University, Taichung, Republic of China; 6 Molecular Medicine Research Center, Chang Gung University, Tao-Yuan, Taiwan, Republic of China; Naval Research Laboratory, United States of America

## Abstract

**Background:**

Bacterial DNA contamination in PCR reagents has been a long standing problem that hampers the adoption of broad-range PCR in clinical and applied microbiology, particularly in detection of low abundance bacteria. Although several DNA decontamination protocols have been reported, they all suffer from compromised PCR efficiency or detection limits. To date, no satisfactory solution has been found.

**Methodology/Principal Findings:**

We herein describe a method that solves this long standing problem by employing a broad-range primer extension-PCR (PE-PCR) strategy that obviates the need for DNA decontamination. In this method, we first devise a fusion probe having a 3′-end complementary to the template bacterial sequence and a 5′-end non-bacterial tag sequence. We then hybridize the probes to template DNA, carry out primer extension and remove the excess probes using an optimized enzyme mix of Klenow DNA polymerase and exonuclease I. This strategy allows the templates to be distinguished from the PCR reagent contaminants and selectively amplified by PCR. To prove the concept, we spiked the PCR reagents with *Staphylococcus aureus* genomic DNA and applied PE-PCR to amplify template bacterial DNA. The spiking DNA neither interfered with template DNA amplification nor caused false positive of the reaction. Broad-range PE-PCR amplification of the 16S rRNA gene was also validated and minute quantities of template DNA (10–100 fg) were detectable without false positives. When adapting to real-time and high-resolution melting (HRM) analytical platforms, the unique melting profiles for the PE-PCR product can be used as the molecular fingerprints to further identify individual bacterial species.

**Conclusions/Significance:**

Broad-range PE-PCR is simple, efficient, and completely obviates the need to decontaminate PCR reagents. When coupling with real-time and HRM analyses, it offers a new avenue for bacterial species identification with a limited source of bacterial DNA, making it suitable for use in clinical and applied microbiology laboratories.

## Introduction

Detection of bacterial DNA holds great promise as a rapid diagnostic tool for early detection of bacterial infections, such as in sepsis [1], [2]. Numerous studies have demonstrated that, among nucleic acid-based methods [3]–[7], broad-range PCR of the conserved bacterial DNA sequences is selective enough to differentiate bacterial from viral and other infections [8]–[10], pointing to the great potential of broad-range PCR in clinical diagnostics of bacterial infection. When coupling with high-resolution melting (HRM) analysis, broad-range PCR can even identify bacteria at the species level [11]–[13], offering a potentially revolutionizing diagnostic platform that saves time and cost and improves diagnostic accuracy.

However, contamination and sensitivity issues have long frustrated efforts to realize the potential of broad-range bacterial DNA amplification in clinical microbiology [14], [15]. In particular, bacterial source DNA contamination in commercially available Taq DNA polymerases has been a challenging problem that, to date, has no satisfactory solution [16], [17]. The contaminating DNA usually include more than one strain or species that cannot be identified as *Thermus aquaticus* or *Escherichia coli* but bear close homology to the species of *Pseudomonas fluorescens*, *Pseudomonas aeruginosa*, *Alcaligenes faecalis*, or *Azotobacter vinelandii* [18]. Because conventional broad-range PCR often co-amplifies these contaminants with the target bacterial DNA, the consequent high false positive rate generally renders accurate interpretation of the results difficult, if not impossible [19].

In the past 20 years, many attempts have been tried to solve this problem. Some examples include UV irradiation, restriction endonuclease digestion, ultrafiltration, and pretreatment of reagent with DNase I [20]–[23]. Unfortunately, all previous attempts either failed to completely eliminate false positives due to inherent limitations on reaction conditions or could not achieve the required level of sensitivity due to apparent inhibition of the PCR reaction [14], [24]. As a result, many laboratories were forced to resort to multiplex PCR or other non-PCR methods that are usually difficult to optimize and perform [25]–[28].

Here we describe and demonstrate an innovative primer extension PCR (PE-PCR) method capable of fully addressing the problem of bacterial DNA contamination in PCR enzymes and reagents. This method can be performed in a single-tube, is highly reproducible, and has sufficient detection limit suitable for use in a clinical and applied microbiology setting. When coupling with real-time and HRM analysis, individual bacterial species can be directly identified from their respective melting profiles without resorting to multiplexing or hybridization probes. We believe that PE-PCR and HRM analysis, together, form a novel molecular diagnostic platform that can potentially revolutionize clinical microbiology laboratory practices.

## Results

### Contamination issue in broad-range amplification of bacterial DNA

**Figure 1:**
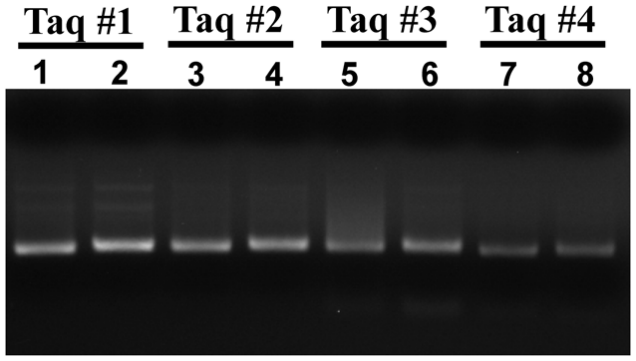
HotStart and low-DNA Taq DNA polymerases are not sufficiently pure for sensitive and specific broad-range amplification of bacterial DNA. The genomic DNA (100 fg) of *S. aureus* was amplified by HotStart or low-DNA Taq DNA polymerases (Taq #1: Hot Start Taq DNA polymerase, Protech Inc.; Taq #2: Fast Hot Start Taq DNA polymerase, KAPA Biosystems; Taq #3: Taq DNA polymerase, TakaRa Inc.; Taq #4: ULTRATOOLS Taq DNA polymerase, Biotools Inc.) using the primer set p201 and p1370 (lanes 1, 3, 5, and 7). Significant amount of PCR product was present in the no template control reactions (lanes 2, 4, 6, and 8).

Several “low-DNA” and HotStart Taq DNA polymerases are available from commercial sources. As a starting point, we first examined 4 commercially available low-DNA or HotStart Taq DNA polymerases to see whether they are suitable for broad-range amplification of bacterial DNA using the universal primer set p201–p1370 [29]. Using these polymerases, we performed classical PCR to amplify a sample containing 100 fg of representative *Staphylococcus aureus* bacterial genomic DNA (equivalent to 20 copies of bacterial genome) and a “no template control (NTC)”. A significant amount of amplified DNA product was found in the NTC reaction, rendering it indistinguishable from the sample reaction (Fig. 1). This result confirms that commercially available Taq DNA polymerase and PCR reagents are not sufficiently pure for broad-range bacterial DNA detection in a clinical setting in which detection limits at the fentogram level is usually required.

### PE-PCR prevents co-amplification of contaminating DNA

Faced with the problem of contamination, all previous attempts had focused on trying to further purify the reagents. This line of thinking represents a frontal assault on the problem that unfortunately turns out to be a futile exercise for the past 20 years. Thinking outside of the box, we sought an alternative route of attack and came up with the PE-PCR strategy.

**Figure 2:**
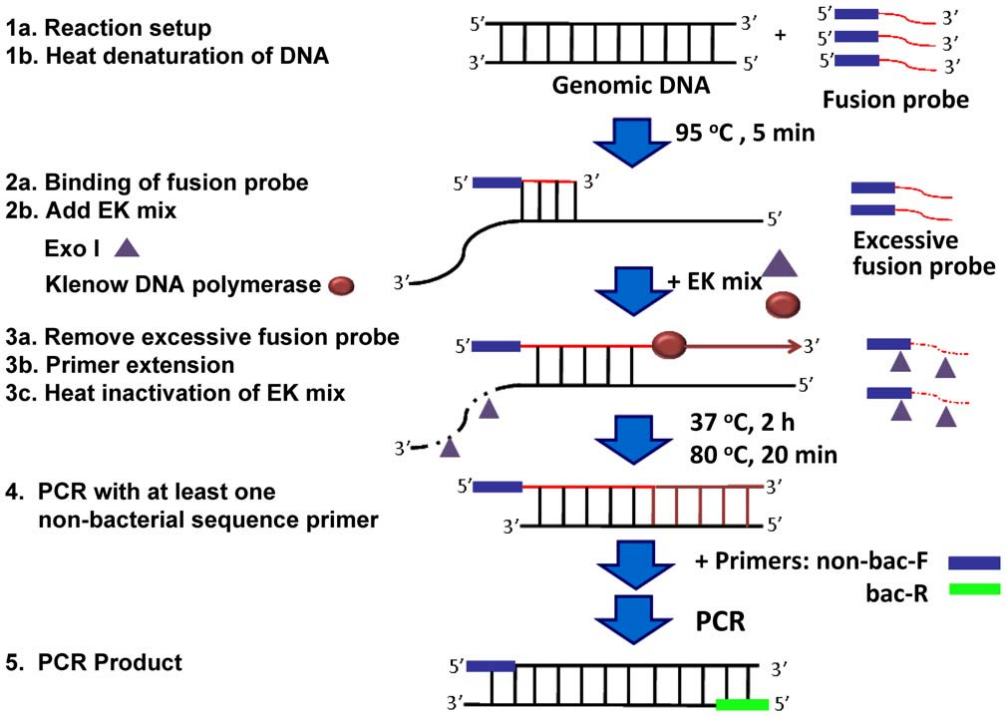
The principle of PE-PCR for bacterial DNA amplification and detection. A fusion probe is designed with the sequences at the 3′-end corresponding to the bacterial genomic sequences and a non-bacterial tag sequence at the 5′-end. The reaction is initiated by annealing the fusion probe to the template bacterial DNA after heat-denaturing at 95°C for 5 min (Step 1 and 2). An enzyme mix (EK mix) of exo I and Klenow DNA polymerase is then added into the reaction mixture and incubated at 37°C for 2 h (Step 3a and 3b). Following heat-inactivation of EK mix at 80°C for 20 min (Step 3c), a forward primer (non-bac-F) corresponding to the non-bacterial sequence of the fusion probe and a reverse primer (bac-R) targeting bacterial genomic sequence downstream of the fusion probe are used for PCR amplification of the primer extension product (Step 4). In this setting, only template bacterial DNA but not the endogenous contaminated bacterial DNA is amplified (Step 5).

The PE-PCR strategy is an approach that combines primer extension and PCR to circumvent the problem of endogenous DNA contamination. As illustrated in Fig. 2, the key component of this strategy is a fusion probe that comprises a bacterial sequence on its 3′-end and a non-bacterial tag sequences on its 5′-end. Using this fusion probe as an extension primer, the PE-PCR reaction was initiated by annealing an excess amount of the fusion probes to the template bacterial DNA after heat-denaturing (Step 1 and 2). An enzyme mix (EK mix) of exonuclease I (exo I) and Klenow DNA polymerase was then added into the reaction mixture. As a result, the unbound/free fusion probes were degraded by exo I and the primer extension reaction was initiated by Klenow DNA polymerase (Step 3a and 3b). Following heat-inactivation of EK mix, a forward primer (non-bac-F) corresponding to the non-bacterial sequence of the fusion probe and a reverse primer (bac-R) targeting bacterial genomic sequence downstream of the fusion probe were used for PCR amplification of the primer-extended product (Step 4). By tagging the template bacterial sequences with a non-bacterial tag sequence prior to PCR amplification (Step 4), the templates are distinguished from the contaminants contained in the PCR reagents. In theory, our PE-PCR strategy should amplify only the tagged template bacterial genomic DNA, thereby, rendering the contaminants a non-issue (Step 5).

**Figure 3:**
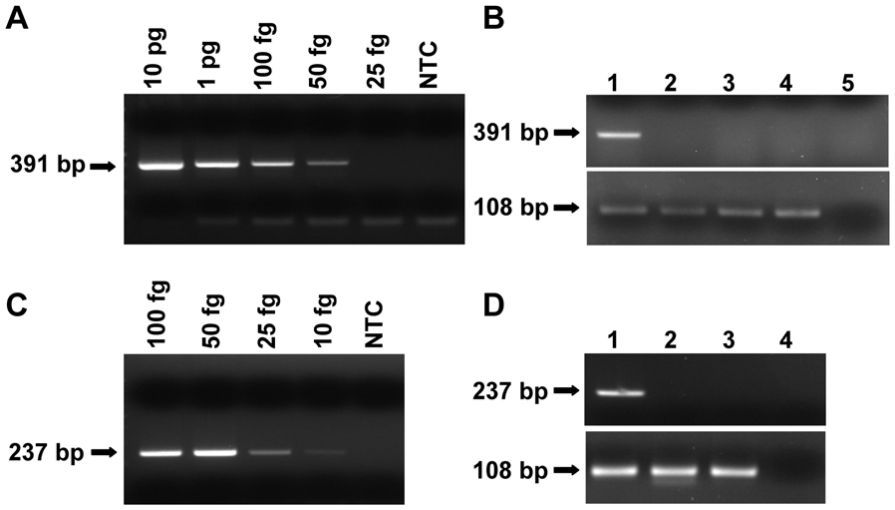
PE-PCR specifically amplifies template bacterial DNA without co-amplification of contaminating bacterial DNA. A. The indicated amount of *S. aureus* genomic DNA was subjected to PE-PCR using the fusion probe M13-TstaG422 and the primer set M13 and TstaG765. B. The *S. aureus* genomic DNA (100 fg) was subjected to PE-PCR (upper panel) as described in panel A (lane 1), in the absence of M13 primer (lane 2), and in the artificially contaminating condition by adding 100 fg *S. aureus* genomic DNA into the EK mix (lane 3) or PCR mixtures (lane 4). PE-PCR was also performed in the absence of template DNA (lane 5). The presence of *S. aureus* genomic DNA was confirmed by species-specific PCR that amplified a chromosomal DNA fragment specific for *S. aureus* (lower panel). C. The indicated amount of *S. aureus* genomic DNA was used as the template for broad-range PE-PCR using the fusion probe M13-16S-p201F and the primer set M13 and p1370. D. The *S. aureus* genomic DNA (100 fg) was subject to broad-range PE-PCR (upper panel) as described in panel C (lane 1), and in the artificially contaminating condition by adding 100 fg of *S. aureus* genomic DNA into the EK mix (lane 2) or PCR mixtures (lane 3). Broad-range PE-PCR was also performed in the absence of template DNA (lane 4). The presence of *S. aureus* genomic DNA was confirmed by species-specific PCR to amplify a chromosomal DNA fragment of *S. aureus* (lower panel). NTC, no template control.

To provide a proof of principle, a serially diluted *S. aureus* genomic DNA was subject to the PE-PCR strategy. The translation elongation factor Tu (Tuf) gene of *S. aureus* was selected as the target for PE-PCR amplification. A fusion probe (M13-TstaG422) was designed with the M13 forward primer sequence at the 5′-end and the Tuf sequences (accession no. AF298796) at the 3′-end (Table 1). After annealing M13-TstaG422 to the template DNA, the EK mix was added into the reaction to degrade the unbound fusion probe and initiate primer extension. The primer extension product was then subject to PCR amplification using M13 and the downstream primer TstaG765 corresponding to the Tuf genomic sequences. As a result, a 391-bp single PCR product was obtained with 50 fg of bacterial DNA equivalent to 10 copies of *S. aureus* genome being detectable by PE-PCR. Notably, no PCR product was observed in the NTC control (Fig. 3A).

**Table 1:** The primers and fusion probes sequences.

Primer/probe type	Primer/probe name	Sequences	Amplicon
***S. aureus*** ** tuf gene (accession number AF298796)**			
Fusion probe	M13-TstaG422	5′-CAGGGTTTTCCCAGTCACGAC GGCCGTGTTGAACGTGGTCAAATC AAAGTTGG-3′	391 bp
Forward primer	M13	5′-CAGGGTTTTCCCAGTCACGAC-3′	
Reverse primer	TstaG765	5′-TAACCATTTCAGTACCTTCTGGTAA-3′	
***S. aureus*** **-specific genomic DNA fragment (accession number AF033191)**			
Forward primer	SA-F	5′-AATCTTTGTCGGTACACGATATTCTTCACG-3′	108 bp
Reverse primer	SA-R	5′-CGTAATGAGATTTCAGTAGATAATACAACA-3′	
**Bacterial 16S rRNA gene**			
Fusion probe	M13-16S-p201F	5′-CAGGGTTTTCCCAGTCACGAC GAGGAAGGTGGGGATGACGTC AAATCATCATG-3′	237 bp
Forward primer	M13	5′-CAGGGTTTTCCCAGTCACGAC-3′	
Reverse primer	p1370	5′-AGICCCGIGAACGTATTCAC-3′	
Forward primer	p201	5′-GAGGAAGGIGIGGAIGACGT-3′	

To mimic bacterial DNA contamination of PCR reagent and enzyme, 100 fg of *S. aureus* genomic DNA was spiked into the EK mix and the Taq DNA polymerase-PCR reaction mixture, respectively. Significant amount of PE-PCR product was generated only when template bacterial DNA was present during primer extension (Fig. 3B, lane 1). No PCR product was generated in the NTC reaction (Fig. 3B, lane 5) or when PCR was performed in the absence of M13 primer (Fig. 3B, lane 2). Remarkably, PE-PCR facilitated amplification of template bacterial DNA without co-amplifying the spiking bacterial DNA (Fig. 3B, lanes 3 and 4). The presence of the spiking DNA in the EK mix or PCR reaction mixture was verified by *S. aureus* species-specific PCR (Fig. 3B, lanes 3 and 4, lower panel). These data indicate no interference from the contaminating bacterial DNA in the PCR reagents and enzymes on specific amplification of the template bacterial DNA and provide a proof of principle for our PE-PCR strategy.

### Broad-range PE-PCR facilitates detection of minute quantities of bacterial DNA

To demonstrate the feasibility of PE-PCR in broad-range amplification of bacterial DNA, we designed a universal fusion probe M13-16S-p201F for broad-range PE-PCR of the 16S rRNA gene that is highly conserved among various bacterial species. Serially diluted *S. aureus* genomic DNA was subjected to broad-range PE-PCR using the fusion probe M13-16S-p201F, the M13 forward primer, the p1370 reverse primer (Table 1), and a routinely used HotStart Taq DNA polymerase (Protech) without applying any decontamination pretreatment. A 237-bp single PCR product was obtained with as little as 10 fg of template DNA equivalent to 2 copies of *S. aureus* genomic DNA being detected by PE-PCR. No PCR product was observed in the NTC reaction (Fig. 3C). Accordingly, the genomic DNAs from a number of clinically important bacterial species have been tested and were shown to be amplifiable by broad-range PE-PCR (data not shown).

We also mimicked bacterial DNA contamination by spiking 100 fg of *S. aureus* genomic DNA into the EK mix and the Taq DNA polymerase-PCR mixture, respectively. As expected, a positive PE-PCR signal was obtained in the presence of 100 fg template bacterial DNA (Fig. 3D, lane 1), whereas no PCR product was generated in the artificially contaminating condition and NTC reaction (Fig. 3D, lanes 2–4). Again, the presence of the spiking DNA in the reaction mixture was verified by *S. aureus* species-specific PCR (Fig. 3D, lower panel). These data together proves that broad-range PE-PCR is capable of fully addressing the problem of bacterial DNA contamination in PCR enzyme and reagents.

To explore the detection limit of this strategy, different concentrations of *S. aureus* genomic DNA ranging from 100 fg to 10 fg were subjected to broad-range PE-PCR. The probability of obtaining a positive PCR signal for 100, 50, 25, and 10 fg of template bacterial DNA was 100%, 95%, 65%, and 55%, respectively (n = 20). Hence, broad-range PE-PCR is easily performed to specifically amplify template bacterial DNA without compromising detection limit and specificity.

### Broad-range real-time PE-PCR couples with HRMA for bacterial species identification

**Figure 4:**
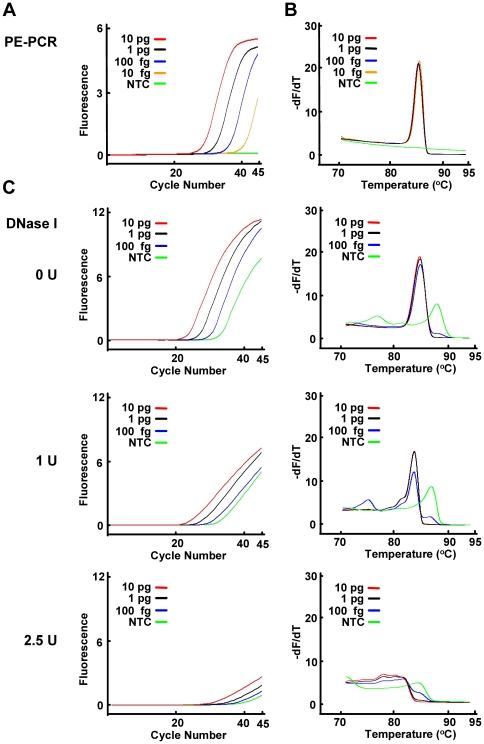
Comparison of broad-range real-time PE-PCR and broad-range real-time PCR with DNase I pretreatment of PCR reagents. A–C. The indicated amounts of *S. aureus* genomic DNA were subject to broad-range real-time PE-PCR using the fusion probe M13-16S-p201F and the primer set M13 and p1370 in the presence of LCGreen I plus HRM dye (panel A and B). Alternatively, the PCR reaction mixtures with or without pretreatment of DNase I (1 U and 2.5 U) were used for broad-range real-time PCR to amplify the indicated amounts of *S. aureus* genomic DNA (panel C). The PCR product was subject to HRM analysis using HR-1 instrument. The amplification (panel A and left panel of C) and derivative plots (panel B and right panel of C) were shown. NTC, no template control.

To further enhance the power of this method, we modified the protocol to incorporate real-time PCR and HRM analysis into the broad-range PE-PCR platform. A 10-fold serially diluted *S. aureus* genomic DNA (10 pg–10 fg) was subject to broad-range real-time PE-PCR in the presence of HRM dye LCGreen I plus. As revealed by the amplification plots, 10 fg of template DNA resulted in amplicon-specific amplification and no PCR product in the NTC reaction (Fig. 4A and 4B). The unique nucleotide contents in the PCR amplicon of *S. aureus* produced a distinctive derivative plot while no melting peak was observed for the NTC reaction. The probability of obtaining a positive PCR signal for 100, 50, 25, and 10 fg of template DNA was 100%, 90%, 50%, and 30%, respectively (n = 10).

For comparison, PCR reagents were pretreated with DNase I followed by broad-range PCR amplification of template *S. aureus* genomic DNA using the primer pair p201 and p1370. As revealed by the amplification and derivative plots (Fig. 4C), the addition of DNase I significantly inhibited PCR amplification. At 1 U of DNase I, the endogenous contaminating DNA was not completely eliminated. Increasing the concentration of DNase I to 2.5 U caused further PCR inhibition and hampered the detection limit fo broad-range PCR amplification of bacterial DNA.

**Figure 5:**
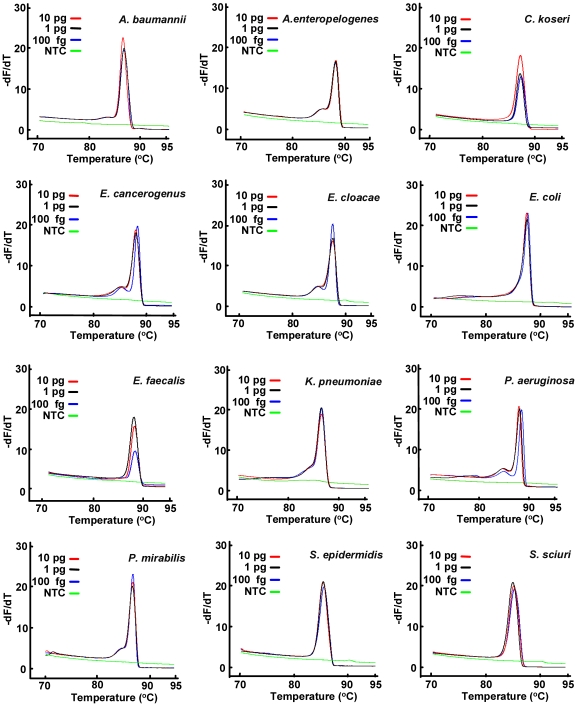
Broad-range real-time PE-PCR and HRM analysis for 12 different bacterial species. The indicated amounts of genomic DNA from 12 different bacterial species were subject to broad-range real-time PE-PCR using the fusion probe M13-16S-p201F and the primer set M13 and p1370 in the presence of LCGreen I plus. The PCR product was subject to HRM analysis using HR-1 instrument and the derivative plots were shown. NTC, no template control.

To further validate our strategy, we applied broad-range real-time PE-PCR to analyze additional 12 common bacterial species (Table 2) using 10-fold serially diluted genomic DNA (10 pg–100 fg). The derivative plots and melting temperature from HRM analyses of the PCR product easily distinguished most of the bacterial species (Fig. 5 and Table 2) without sequencing of the PCR amplicon.

**Table 2:** High-resolution melting profiles for clinically important bacterial species disclosed by broad-range PE-PCR of 16S rRNA gene.

Bacterial species (n)^a^	T_m_ ± SD (°C)	GC content (%)	GenBank accession no.
*S. sciuri* (5)	85.08±0.30	48.1	AB233332
*S. epidermidis* (3)	85.15±0.31	48.1	L37605
*S. aureus* (5)	85.25±0.31	48.5	Y15856
*K. pneumoniae* (3)	86.42±0.27	52.3	AF511429
*P. mirabilis* (3)	86.64±0.19	52.3	AF008582
*C. koseri* (5)	86.71±0.21	52.7	EF059880
*A. baumannii* (3)	87.00±0.11	54.9	AY738399
*E. cloacae* (6)	87.56±0.21	54.0	DQ089673
*E. coli* (4)	87.66±0.04	54.9	AF511430
*E. cancerogenus* (4)	87.71±0.20	53.8	EF011116
*E. faecalis* (6)	87.84±0.23	54.4	AB292313
*P. aeruginosa* (3)	87.90±0.21	54.8	Z76672
*A. enteropelogenes* (3)	88.26±0.04	54.8	EF465529

a Number of test.

## Discussion

Bacterial DNA detection in a testing sample is crucial for clinical, environmental and applied microbiology. Despite the great potential of broad-range PCR of the conserved bacterial DNA sequences as a molecular diagnostic tool [30], [31], its application has been hampered by the difficulty in removing contaminating bacterial DNA present in Taq DNA polymerase and PCR reagents. The lack of reliable methods for decontaminating the PCR reagents has long challenged the field. The PE-PCR technique we report in this study provides a simple and elegant solution to this long standing problem and promises the diagnostic potential of broad-range PCR detection.

Unlike previous attempts to solve the contamination problem, our approach cleverly side-steps the problem by tagging the templates with a non-bacterial sequence using a fusion probe for primer extension. The key factors important to successful PE-PCR hinges on the design of fusion probes and the appropriate ratio of EK mix to ensure proper primer extension and removal of unbound/free fusion probe. Our strategy offers several advantages compared with previous methods. Most of the previously reported methods are based on DNA degradation strategy including the use of DNase I, UV irradiation, exonuclease III and restriction endonuclease to eliminate contaminating bacterial DNA [20]–[23], [32]–[34]. Among these methods, adding DNase I into the PCR enzyme mixes followed by heat inactivation is the most classical and popular approach. However, we and others noted that successful removal of contaminants is dosage-dependent with respect to the amount of DNase I [8], [14]. The optimized working concentration of DNase I varies between laboratories, is difficult to optimize, and is usually in a narrow range [14]. The addition of DNase I also has the undesirable effect on inhibition of PCR reaction. In contrast, our strategy is designed to obviate the need for decontamination. Because our strategy does not alter the PCR reagents, it does not cause PCR inhibition or template DNA degradation. Indeed, our data demonstrate that artificial contamination by spiking bacterial DNA into the reagents or Taq DNA polymerase does not affect PE-PCR-mediated amplification of template bacterial DNA.

Moreover, our strategy does not result in any compromise of the detection limit. In the case of bacteremia, it is estimated that approximate 10–15 bacteria are present in 1 ml of patient blood [35]. In practice, about 1–3 ml of blood are usually used as starting material for molecular diagnostics of bacterial infection. Therefore, a detection limit equivalent to about 100 fg or 20 copies of bacterial DNA is required. As found in our analysis (Fig. 3 and 4), the detection limit for broad-range PE-PCR can reach 10 fg (equivalent to 2 copies of bacterial DNA) with roughly 55% of probability. At 100 fg, the probability of detection is 100%. This level of sensitivity should be sufficient for detection of bacterial infection in most clinical specimens.

With an eye towards actual clinical implementation, our broad-range PE-PCR can be dovetailed with a variety of other analytical methods to identify individual bacterial species. As an example, we demonstrated the integration of the broad-range PE-PCR protocol with real-time PCR and HRM analyses to form a complete analytical platform for applications such as quantification of pathogen load and/or species identification [11]. Similar to our previous findings, HRM analyses of broad-range real-time PE-PCR product results in distinguishable HRM profiles and generates unique molecular fingerprints for various bacterial species. In this study, the molecular fingerprints for 13 different bacterial species have been established. These profiles can be expanded further by including additional bacterial species. With a comprehensive database of HRM profiles, identification of bacterial species can be automated by scanning the molecular fingerprint between the unknown sample and the standard in the database. If required, heteroduplex formation or multiple PCR fragments can be employed to distinguish the bacterial species with closely similar HRM profile. These strategies have been reported by a number of investigations for microorganism identification [11]–[13], [36]–[39] and add to the power of our broad-range PE-PCR.

In spite of the conceptual elegance and superior performance of our strategy, there is still one potential weakness that may potentially derail our method. During the step in which the fusion probe is added, the templates are not yet tagged, hence, they are not yet protected from the effects of contamination. Thus, care must be taken in handling of the samples during the steps of probe adding and primer annealing so to avoid contamination from the working environment. Once the templates are tagged, the contamination that occurs thereafter is not likely to cause a false positive signal. This notion is supported by our data demonstrating that no PCR product was observed in the artificial contamination condition with bacterial DNA being spiked into the EK mix or PCR reagents. Hence, PE-PCR greatly simplifies the procedure of DNA decontamination and reduces the risk of contamination during broad-range amplification of bacterial DNA.

Similar to other broad-range amplification techniques, species identification by HRM analysis may be compromised by multiple infections that account for 5%–22% of cases of bacteremia [35], [40], [41]. These clinical complexities may not be crucial when bacterial isolates are used for broad-range PCR amplification but may limit the capability of PE-PCR for detecting multiple bacterial species when PCR is performed directly with clinical specimens. Nevertheless, the HRM parameters such as the presence of multiple melting peaks in the derivative plot may provide information for differentiating single vs. multiple infections in clinical specimens. In addition, the combination of multiple species-specific unlabeled probes [42] with HRM can be explored for bacterial species identification when patients are co-infected with multiple types of bacteria.

In conclusion, our PE-PCR based methods provide a turnkey solution that solves the long standing problem of endogenous bacterial DNA contamination. The broad-range PE-PCR is fast, reproducible, highly specific and extremely sensitive, and can be conveniently combined with the commercially available bacterial DNA extraction technique in an automated or semi-automated fashion. Because broad-range PE-PCR renders the presence of endogenous bacterial DNA in the PCR reagents and Taq DNA polymerase a non-issue during PCR amplification of minute sample DNA, this method can have a broader reach beyond bacterial DNA amplification and detection. We fully expect that the broad-range PE-PCR amplification system is also useful for mycobacterium and fungi detection that encounters similar challenge of reagent contamination [3]. When adapting to real-time PCR and HRM platforms, this method provides a new avenue for microbial identification in clinical and applied microbiology laboratories.

## Materials and Methods

### Materials

The exo I and Klenow DNA polymerase were purchased from New England Biolab (Ipswich, MA). The LCGreen I plus reagent set and HR-1 instrument were purchased from Idaho Technology (Salt Lake City, UT). The HotStart Taq DNA polymerase was purchased from Protech (Taipei, Taiwan). The Fast Hot Start Taq DNA polymerase was purchased from KAPA Biosystems (Woburn, MA). The “low-DNA” Taq DNA polymerase was purchased from Takara (Shiga, Japan). The ULTRATOOLS Taq DNA polymerase was purchased from Biotools Inc. (Madrid, Spain). The LightCycler capillaries were purchased from Roche Applied Science (Indianapolis, IN). The DNase I was purchased from Promega (Madison, WI). Bacterial strains were clinical isolates as described previously [11]. A complete list of primer and fusion probe sequences is shown in Table 1.

### Isolation of bacterial genomic DNA

The overnight culture bacterial suspension (4 ml) was centrifuged at 10,000 rpm for 10 min and the pellet was resuspended in 4 ml of solution I buffer (25 mM Tris-HCl, pH 7.5, 50 mM glucose, 10 mM EDTA, and 40 µg/ml lysostaphin). The bacterial suspension was incubated at 37°C for 2 h and the reaction buffer containing 280 µl of 20% SDS and 40 µl of proteinase K (10 mg/ml) and RNase A (10 mg/ml) was added to the bacterial suspension and incubated at 55°C overnight. After phenol/chloroform extraction, supernatant was transferred to a clean microcentrifuge tube, and the DNA was precipitated with ethanol. After washing twice with 75% ethanol, DNA pellets were resuspended in water for quantification and the subsequent PCR assays.

### PE-PCR

Conventional PE-PCR was performed within one reaction tube. Briefly, the annealing step consisted of a 20 µl annealing mix containing 8 µl of H_2_O, 5 µl of fusion probe (2 ng/µl), 5 µl of bacterial genomic DNA at the indicated concentration, and 2 µl of 10× PCR buffer. The reaction mixture was heated to 95°C for 5 min and was kept at 37°C. Then 11 µl of EK mix consisting of 3 µl of H_2_O, 1 µl of 10× PCR buffer, 5 µl of dNTP (2 mM), 1 µl of Klenow DNA polymerase (5 U/µl), and 1 µl of exo I (20 U/µl) was added to the annealing mix and incubated at 37°C for 2 h. After heat inactivation at 80°C for 20 min, the reaction mixture was brought up to 50 µl by adding 14 µl of H_2_O, 2 µl of 10× PCR buffer, 1 µl of forward primer M13 (5 µM), 1 µl of reverse primer (5 µM), and 1 µl of HotStart Taq DNA polymerase (5 U/µl). The PCR cycling condition was 1 cycle of 95°C for 10 min, 45 cycles of 95°C for 15 s, and 60°C for 1 min. For detection of spiking *S. aureus* genomic DNA, the M13 and the reverse primer was replaced by the primer set of SA-F and SA-R (Table 1) that specifically amplifies *S. aureus* genomic DNA fragment [43].

For real-time PE-PCR, the reaction was proportionally scaled down to 8 µl during binding of fusion probe and primer extension. Then the reaction mixture was brought up to 20 µl by adding 1.5 µl of H_2_O, 2 µl of 10× bovine serum albumin (BSA), 2 µl of 10× LCGreen I plus, 0.8 µl of 10× PCR buffer, 0.4 µl of forward primer M13 (5 µM), 0.4 µl of reverse primer (5 µM), and 0.5 µl of HotStart Taq DNA polymerase (5 U/µl) and was transferred to the capillary tube. Real-time PCR was performed using LightCycler 1.5 instrument and the cycling condition was 1 cycle of 95°C for 10 min, 45 cycles of 95°C for 15 s, and 60°C for 1 min at a transition rate of 20°C/s.

### High-resolution melting curve acquisition and analysis

Glass capillaries containing amplification products were transferred to the HR-1 instrument (Idaho Technology) and the PCR fragments were melted at 64–96°C at a rate of 0.3°C/s. Melting profiles such as the derivative plots and melting temperatures were assessed with HR-1 software.

### Pretreatment of DNase I for decontamination and broad-range PCR amplification

The PCR reagents containing 2 µl of 10× BSA, 2 µl of dNTP (2 mM), 2 µl of 10× LCGreen I plus, 1 µl of 10× PCR buffer, 0.5 µl of HotStart Taq DNA polymerase (5 U/µl), were incubated with DNase I (1 U or 2.5 U) at 37°C for 30 min. After heat inactivation of DNase I at 85°C for 15 min, the reaction mixture was brought up to 20 µl by adding 1 µl of 10× PCR buffer, 2 µl of forward primer p201 (5 µM), 2 µl of reverse primer p1370 (5 µM), and 5 µl of template DNA. The PCR cycling condition was 1 cycle of 95°C for 10 min, 45 cycles of 95°C for 15 s, and 60°C for 1 min at a transition rate of 20°C/s.
